# COX‐2/PGE_2_ axis regulates hippocampal BDNF/TrkB signaling via EP2 receptor after prolonged seizures

**DOI:** 10.1002/epi4.12409

**Published:** 2020-06-09

**Authors:** Ying Yu, Jianxiong Jiang

**Affiliations:** ^1^ Department of Pharmaceutical Sciences College of Pharmacy University of Tennessee Health Science Center Memphis TN USA

**Keywords:** CREB, epilepsy, epileptogenesis, neurotrophin, seizure, status epilepticus

## Abstract

**Objective:**

The objective of this study was to identify the signaling pathway that is immediately triggered by status epilepticus (SE) and in turn contributes to the excessive brain‐derived neurotrophic factor (BDNF)/tropomyosin‐related kinase receptor B (TrkB) signaling within the hippocampus.

**Methods:**

We used quantitative PCR, enzyme‐linked immunosorbent assay, and Western blot analysis to examine gene expression at both mRNA and protein levels in the hippocampus following prolonged SE in mice and rats. Three classical animal models of SE were utilized in the present study to avoid any model‐ or species‐specific findings.

**Results:**

We showed that both cyclooxygenase‐2 (COX‐2) and BDNF in the hippocampus were rapidly upregulated after SE onset; however, the induction of COX‐2 temporally preceded that of BDNF. Blocking COX‐2 activity by selective inhibitor SC‐58125 prevented BDNF elevation in the hippocampus following SE; prostaglandin E2 (PGE_2_), a major product of COX‐2 in the brain, was sufficient to stimulate hippocampal cells to secrete BDNF, suggesting that a PGE_2_ signaling pathway might be directly involved in hippocampal BDNF production. Inhibiting the Gα_s_‐coupled PGE_2_ receptor EP2 by our recently developed selective antagonist TG6‐10‐1 decreased the SE‐triggered phosphorylation of the cAMP response element‐binding protein (CREB) and activation of the BDNF/TrkB signaling in the hippocampus.

**Significance:**

The molecular mechanisms whereby BDNF/TrkB signaling is upregulated in the hippocampus by SE largely remain unknown. Our findings suggest that COX‐2 via the PGE_2_/EP2 pathway regulates hippocampal BDNF/TrkB activity following prolonged seizures. EP2 inhibition by our bioavailable and brain‐permeable antagonists such as TG6‐10‐1 might therefore provide a novel strategy to suppress the abnormal TrkB activity, an event that can sufficiently trigger pathogenic processes within the brain including acquired epileptogenesis.

**Key Points**

- The induction of COX‐2 temporally and quantitatively leads that of BDNF in the hippocampus after SE
- Selective COX‐2 inhibition blocks BDNF elevation in the hippocampus following SE
- PGE_2_ alone is able to stimulate hippocampal cells to produce BDNF
- Inhibiting PGE_2_ receptor EP2 decreases SE‐triggered phosphorylation of the transcription factor CREB and TrkB receptor in the hippocampus
- EP2 receptor might provide a novel molecular target to suppress the abnormal TrkB activity after prolonged seizures

## 1 INTRODUCTION

As the fourth most common neurological disorder, epilepsy afflicts about 1% of the global population at all ages. In spite of marked advances in epilepsy treatment during the past few decades, there are still more than 30% of epilepsy patients showing considerable pharmacoresistance to current therapies^1^; current antiseizure drugs (ASDs) are also well known for their broad neurotoxic adverse effects, such as dizziness, drowsiness, headache, irritability, nausea, and changes in emotion, behavior, and cognition.^2^ It is another very disappointing fact that the current ASDs merely provide symptomatic relief, as no US FDA‐approved drug has yet been demonstrated to prevent the disease or modify its progression. Developing new antiepileptic and potentially antiepileptogenic therapeutics for this debilitating condition is an urgent unmet need.^3^, ^4^ A major obstacle to identifying novel antiepileptic and potentially antiepileptogenic targets is that the molecular mechanisms whereby normal brains are transformed to generate epileptic seizures after various initial precipitating incidents—such as head injuries, strokes, brain tumors, and status epilepticus (SE)—remain unsolved.

Commonly considered criteria for potential molecular targets for disrupting epileptogenesis include the requirements that their expression is dynamically regulated by seizures and that such an expression change is necessary and sufficient to cause structural and functional alterations in the brain that lower the seizure threshold. As such, the brain‐derived neurotrophic factor (BDNF) has emerged as such an attractive candidate target over the past two decades.^5^, ^6^ BDNF via its tropomyosin‐related kinase receptor B (TrkB) regulates a variety of physiological processes such as learning, memory, and reward.^7^ However, the excessive activation of TrkB receptor by BDNF in adult brains has been proposed requisite for epileptogenesis following prolonged seizures. Both BDNF and TrkB are upregulated after epileptic seizures in experimental animals and patients with temporal lobe epilepsy (TLE).^8^, ^9^, ^10^, ^11^, ^12^ Intracerebroventricular infusion of antibodies of TrkB, but not TrkA or TrkC, blocks kindling development in rats.^13^ Likewise, the increased TrkB signaling within the hippocampal mossy fiber pathway and CA3 stratum lucidum region is related to seizure induction in mouse kindling model.^14^, ^15^ The downstream phosphoinositide‐specific phospholipase C‐γ1 (PLC‐γ1) seems essential to BDNF/TrkB‐mediated epileptogenesis, as a TrkB mutation designed to uncouple PLC‐γ1 from TrkB was able to impair the kindling development in mice.^16^ Moreover, inhibiting TrkB kinase activity by the mutant kinases inhibitor II or dissociating PLC‐γ1 from TrkB via a competitive peptide prevented the seizure recurrence and anxiety‐like comorbidity in mice after kainic acid‐induced SE.^17^, ^18^

It appears that the BDNF receptor TrkB and its downstream effector PLC‐γ1 provide promising molecular targets for the prevention or suppression of acquired epilepsy of various etiologies.^19^, ^20^ However, a key unsolved puzzle is the upstream signaling events that are immediately triggered by acute seizures and directly promote hippocampal BDNF/TrkB activation after SE, thereby causing epileptogenesis. The inducible cyclooxygenase (COX) isoform, COX‐2, has been reported to regulate the BDNF expression in the hippocampus after systemic administration of a low dose of kainic acid (6‐12 mg/kg, ip) in rats.^21^ However, the role for COX‐2 cascade in BDNF/TrkB signaling following prolonged SE remains to be determined. In the present study, we used three classical animal models of SE to investigate the effects of COX‐2 inhibition on BDNF expression in the hippocampus following SE. Taking advantage of a novel, bioavailable, and brain‐permeable small‐molecule antagonist that we recently developed to suppress inflammation in the CNS, this study was also aimed to identify the downstream prostaglandin E2 (PGE_2_) receptor subtype that is involved in the regulation of BDNF/TrkB signaling within the hippocampus after prolonged seizures.

## 2 MATERIAL AND METHODS

### 2.1 Chemicals and drugs

Methylscopolamine, terbutaline, pilocarpine, and sodium pentobarbital were purchased from Sigma‐Aldrich. SC‐58125 and kainic acid were purchased from Tocris Bioscience. 16,16‐dimethyl prostaglandin E2 (dmPGE_2_) was purchased from Cayman Chemical. Diazepam was purchased from Henry Schein. Compound TG6‐10‐1 was synthesized accordingly,^22^ and the purity was confirmed by LC/MS and NMR in the Medicinal Chemistry Core at the University of Tennessee Health Science Center.

### 2.2 Experimental animals

Animal procedures were approved by the Institutional Animal Care and Use Committee (IACUC) of the University of Tennessee Health Science Center and performed in accordance with the Guide for the Care and Use of Laboratory Animals (the Guide) from the NIH. Adult male C57BL/6 mice and Sprague Dawley rats were housed under a 12‐hr light/dark cycle with food and water ad libitum.

### 2.3 Mouse pilocarpine model of SE

Adult male mice (8‐10 weeks) were injected with methylscopolamine and terbutaline (2 mg/kg each in saline, ip) to minimize the unwanted effects of pilocarpine in the periphery. Thirty minutes later, pilocarpine (280 mg/kg in saline, freshly prepared, ip) was injected to induce seizures in mice. Control mice received methylscopolamine and terbutaline, followed by saline injection instead of pilocarpine. Seizures were classified as we previously described.^22^, ^23^ 0: normal behavior—walking, exploring, sniffing, and grooming; 1: immobile, staring, jumpy, and curled‐up posture; 2: automatisms—repetitive blinking, chewing, head bobbing, vibrissae twitching, scratching, face‐washing, and “star‐gazing”; 3: partial body clonus, occasional myoclonic jerks, and shivering; 4: whole body clonus, “corkscrew” turning and flipping, loss of posture, rearing, and falling; 5: (SE onset): nonintermittent seizure activity; 6: wild running, bouncing, and tonic seizures; and 7: death. SE was defined by nonintermittent seizure activity, which was indicated by continuous generalized clonic seizures without returning to lower‐stage seizures. The SE was allowed to proceed for 1 hr and terminated by sodium pentobarbital (30 mg/kg, ip). The animal mortality rate after pilocarpine‐induced SE was about 40% in this study.

### 2.4 Rat pilocarpine model

Adult male rats (~250 g) were pretreated with methylscopolamine and terbutaline (2 mg/kg each, ip) to reduce the peripheral effects of pilocarpine. Thirty minutes later, rats received a single dose of pilocarpine (350 mg/kg, ip) for seizure induction; the behavioral seizures were classified accordingly. SE was defined by uninterrupted seizure activity (stage 5), allowed to proceed for 90 minutes, and terminated by sodium pentobarbital (30 mg/kg, ip). A total of 10 rats that survived pilocarpine‐induced SE were treated by vehicle or COX‐2 inhibitor SC‐58125 in this study. In addition, 10 saline‐treated rats were used in control groups.

### 2.5 Mouse kainic acid model

Adult male mice (8‐10 weeks) were treated by kainic acid (30 mg/kg, ip) to induce seizures, which were classified as we previously described.^24^, ^25^ 0: normal behavior—walking, exploring, sniffing, and grooming; 1: arrest and rigid posture; 2: head bobbing; 3: partial body clonus (unilateral forelimb clonus), myoclonic jerk, and lordotic posture; 4: rearing with bilateral forelimb clonus; 5: rearing and falling (loss of postural control); 6: tonic‐clonic seizure with running and jumping; and 7: death. The onset of behavioral SE was defined as the occurrence of three stage‐4 seizures. SE was interrupted by diazepam (10 mg/kg, ip) 1 hr after SE began. A total of 40 mice were treated with kainic acid, and 21 of them experienced SE. The 19 mice that did not enter SE were removed from the study; two mice experienced SE died during the post‐SE recovery.

### 2.6 Drug treatments

Two hours after SE onset, that is, 0.5‐1 hr after sodium pentobarbital or diazepam was administered, animals were randomized for treatment twice daily: Rats were treated with COX‐2 inhibitor SC‐58125 (3 mg/kg, ip); mice were treated with EP2 antagonist TG6‐10‐1 (5 mg/kg, ip). During recovery from SE, animals were fed moistened rodent chow, monitored daily, and injected with 5% dextrose in lactated Ringer's solution (Baxter) when necessary. Animals were then euthanized under deep anesthesia with isoflurane and perfused with ice‐cold phosphate‐buffered saline (PBS) to wash blood out of the brain. Animal brains were dissected out, and the hippocampal tissues were collected for biochemical analyses.

### 2.7 Quantitative PCR

The mRNA levels of interested genes in the hippocampus were quantified by quantitative PCR (qPCR) as previously described.^24^, ^26^, ^27^ The sequences of primers for qPCR were as follows: GAPDH, forward 5′‐TGTCCGTCGTGGATCTGAC‐3′ and reverse 5′‐CCTGCTTCACCACCTTCTTG‐3′; COX‐2, forward: 5′‐CTCCACCGCCACCACTAC‐3′ and reverse: 5′‐TGGATTGGAACAGCAAGGAT‐3′; mPGES‐1, forward: 5′‐ATCAAGATGTACGCGGTGGC‐3′ and reverse: 5′‐GAGGAAATGTATCCAGGCGA‐3′; BDNF, forward: 5′‐GCCGCAAACATGTCTATGAGGGTT‐3′ and reverse 5′‐TTGGCCTTTGGATACCGGGACTTT‐3′.

### 2.8 Western blot analysis

Western blot analysis was performed to measure the protein expression of interested genes in brain samples as previously described.^28^ The primary antibodies used in this study were as follows: rabbit anti‐COX‐2 (1:1,000, Abcam, #ab15191), rabbit anti‐BDNF (1:750, Santa Cruz Biotech, #sc‐546), rabbit anti‐EP2 (1:1,000, Cayman Chemical, #101750), rabbit anti‐CREB (1:1,000, Millipore, #AB3006), rabbit anti‐p‐CREB (pS133) (1:1,000, Millipore, #AB3442), rabbit anti‐TrkB (1:1,000, Millipore, #07‐225), rabbit anti‐p‐TrkB (pY816) (1:1,000, Abcam, #ab81288), goat anti‐p‐TrkB (pY705/706) (1:1,000, Santa Cruz Biotech, #sc‐7996), and mouse anti‐GAPDH (1:5,000, Calbiochem #CB1001). The Western blot band intensity was quantified using ImageJ (NIH).^24^ The protein expression level of a specific gene was first normalized to the loading control GAPDH and then to the mean of control group for comparisons.

### 2.9 Primary hippocampal cells

Hippocampal cells were isolated from embryos (E18) of timed‐pregnant Sprague Dawley rats as we previously described.^25^ The cells were seeded onto poly‐D‐lysine (Sigma‐Aldrich) coated 24‐well plates at a density of 150 000 cells/well in Neurobasal medium supplemented with B27, 5% fetal bovine serum (FBS), 100 U/mL penicillin, and 100 µg/mL streptomycin (Invitrogen). Cells were incubated at 37°C in a humidified atmosphere consisting of 5% CO_2_ and 95% air, and half of the culture medium was replaced twice a week with Neurobasal medium without FBS. The majority of the cultured cells were hippocampal neurons, confirmed by immunocytochemistry. After 14 days, hippocampal cells were treated by dmPGE_2_ overnight, and the BDNF in the culture medium was measured by enzyme‐linked immunosorbent assay (ELISA). It is noted that the BDNF in the fresh complete culture medium was undetectable.

### 2.10 PGE_2_ and BDNF measurement

The PGE_2_ and BDNF levels in the culture medium and the hippocampal tissues of rats and mice after SE were measured using PGE_2_ Multi‐Format ELISA Kit (Arbor Assays, #K051) and BDNF E_max_ ImmunoAssay System (Promega, #TB257), respectively. A 50 µL of diluted culture medium or tissue lysate was used for each PGE_2_ and BDNF measurement according to the manufacturer's protocols and as we previously described.^27^, ^28^ The OD generated from each well was measured using a Synergy H1 microplate reader (BioTek) at 450 nm. A standard curve for PGE_2_ or BDNF was run with each experiment. The PGE_2_ or BDNF levels were normalized to mean of the control group for comparisons.

### 2.11 Statistical analysis

Statistical analyses were performed using GraphPad Prism 7.05 by *t* test, one‐way, or two‐way analysis of variance (ANOVA) with post hoc Dunnett's or Bonferroni's multiple comparisons test as indicated. Correlation analyses were performed using Pearson correlation coefficient. Grubbs' test was utilized to identify the outliers, and none was found in this study. *P* < .05 was considered statistically significant. Data are presented as mean ±/+ SEM.

## 3 RESULTS

### 3.1 Hippocampal BDNF synthesis is correlated with and preceded by COX‐2 induction following SE

**FIGURE 1:**
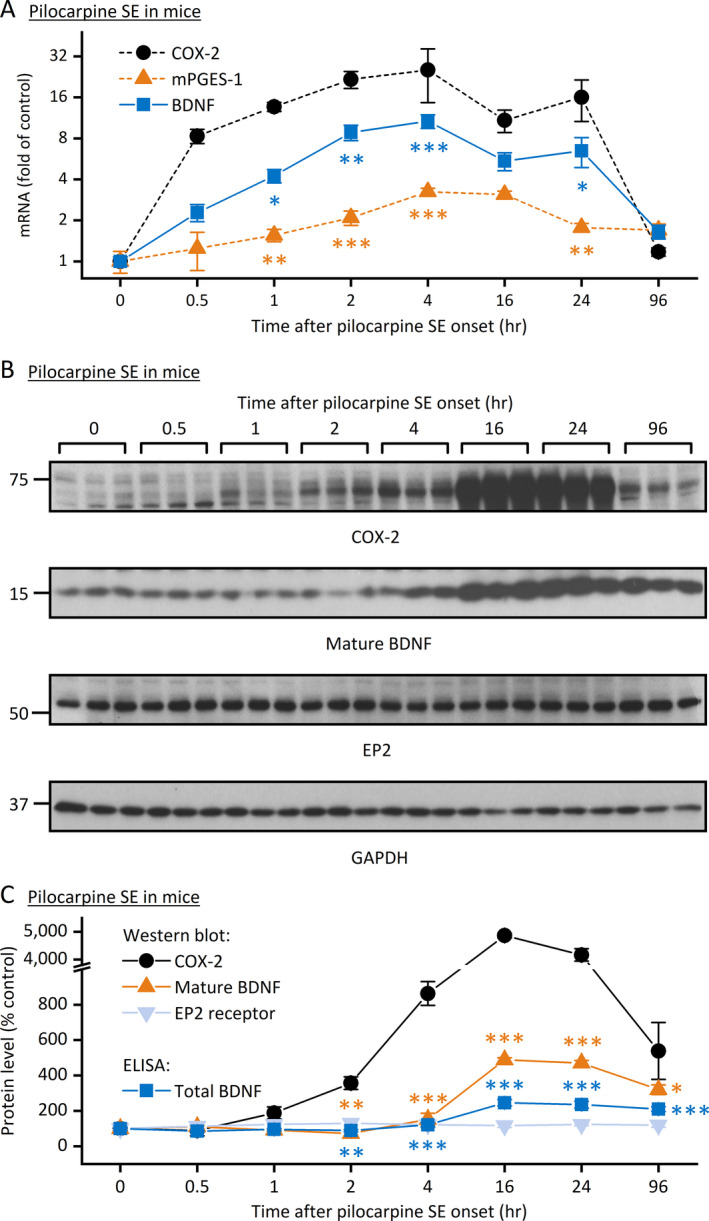
Correlative expression of COX‐2 and BDNF following pilocarpine SE in mice. A, Quantitative PCR (qPCR) was performed to measure the mRNA levels of COX‐2, mPGES‐1, and BDNF in the hippocampus after pilocarpine‐induced SE in mice. The mRNA levels of all three tested genes were increased in a time‐dependent manner; the mRNA induction of COX‐2 was quicker and higher than mPGES‐1 and BDNF (N = 6‐8, *P* < .001 for both COX‐2 vs mPGES‐1 and COX‐2 vs BDNF, *P < .05; **P < .01; ***P < .001 for comparison to COX‐2 at individual time points, two‐way ANOVA and post hoc Dunnett's multiple comparisons test). B, The protein levels of COX‐2, EP2, and BDNF (mature form) in the hippocampus after pilocarpine SE in mice were assessed by Western blot analysis with GAPDH as the loading control. Three representative samples from each time point after SE onset are shown on the blots. C, The immunoblots of COX‐2, mature BDNF, and EP2 were scanned, and the band intensities were quantified using ImageJ. The total BDNF protein levels (mature and proform) in the hippocampus were measured by ELISA. The relative protein expression levels of COX‐2, BDNF, and EP2 were normalized to their basal levels (0 h after SE onset). Both COX‐2 and BDNF (mature and total) were rapidly and substantially elevated in the hippocampus after pilocarpine SE in mice; the protein induction of COX‐2 was quicker and higher than both mature and total BDNF (N = 6, *P* < .001 for both COX‐2 vs mature BDNF and COX‐2 vs total BDNF, *P < .05; **P < .01; ***P < .001 for comparison to COX‐2 at individual time points, two‐way ANOVA and post hoc Dunnett's multiple comparisons test). Data are shown as mean ± SEM

We previously reported that the COX‐2 enzyme was immediately and robustly induced to synthesize PGE_2_ in the brain following prolonged seizures.^29^, ^30^ It is well known that the BDNF signaling via TrkB receptor is enhanced within the hippocampus by SE and is believed to contribute to the development of acquired epilepsy.^17^, ^18^ To investigate the temporal sequence of induction for COX‐2 signaling cascade and BDNF/TrkB pathway, we first examined the mRNA levels of COX‐2, the membrane‐associated PGE synthase‐1 (mPGES‐1) that directly synthesizes PGE_2_ from COX‐2‐derived PGH_2_, and BDNF in the hippocampus following pilocarpine‐induced SE in mice. We found that the mRNA levels of all these three examined genes in the hippocampus were substantially increased in SE mice in a time‐dependent manner when compared to the control cohorts (Figure 1A). Though all three genes began to show noticeable induction as early as 30 minutes after SE began, the COX‐2 expression level was significantly higher than that of mPGES‐1 or BDNF throughout the induction course (Figure 1A). The leading induction of COX‐2 expression was further confirmed by Western blot analysis (Figure 1B), which revealed that COX‐2 protein was noticeable elevated 1 hour after SE onset and began to show substantial increase another hour later. In contrast, the mature form of BDNF was not induced until 4 hour after SE and its induction level was significantly lower than that of COX‐2 (Figure 1C). The delayed and lower induction of BDNF—compared to COX‐2—was also confirmed by ELISA, which was used to measure the total BDNF proteins consisting of both mature BDNF and its precursor (Figure 1C). Interestingly, the induction of total BDNF was even lower than the mature BDNF (Figure 1C), indicating that the mature BDNF was the major form that was induced in the hippocampus following SE. These results from qPCR and ELISA together suggest that the induction of COX‐2 temporally and quantitatively led that of BDNF in the hippocampus following SE.

### 3.2 COX‐2 activity and PGE_2_ signaling are involved in seizure‐promoted hippocampal BDNF production

**FIGURE 2:**
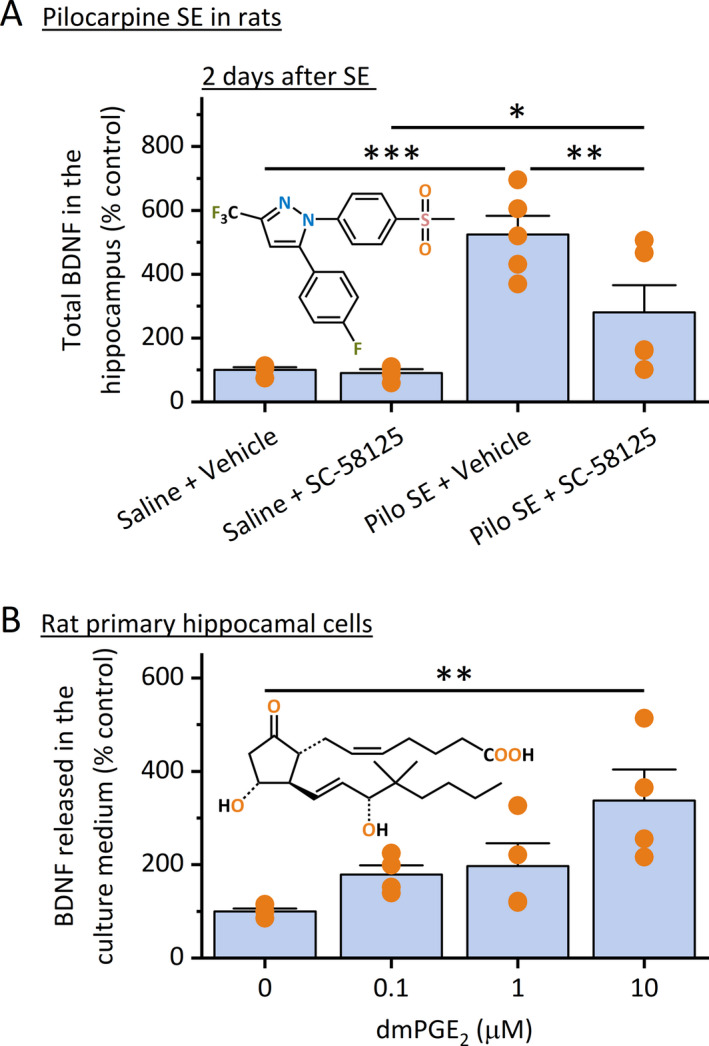
COX‐2 and PGE_2_ are involved in BDNF production after pilocarpine SE in rats. A, Rats were injected with pilocarpine (350 mg/kg, ip) for the induction of SE, which proceeded for 90 min and was terminated by sodium pentobarbital (30 mg/kg, ip). Rats were then treated with COX‐2 inhibitor SC‐58125 (3 mg/kg, sc) twice daily for two days. The total BDNF (both proform and mature form) in the hippocampus was measured by ELISA. Treatment with SC‐58125 decreased seizure‐induced hippocampal BDNF (N = 5, *P < .05; **P < .01; ***P < .001, one‐way ANOVA with post hoc Bonferroni's multiple comparisons test). B, Rat primary hippocampal cultures were treated with dmPGE_2_ overnight, and the BDNF levels in the culture medium were measured by ELISA. Note that dmPGE_2_ treatment increased BDNF levels secreted by these cells in a concentration‐dependent manner (N = 4, **P < .01 compared to control, one‐way ANOVA with post hoc Bonferroni's multiple comparisons test). Data shown as mean + SEM. The chemical structures of SC‐58125 and dmPGE_2_ are shown in the insets

We next wanted to determine whether COX‐2 induction is necessary for BDNF synthesis following SE. SE was induced in rats by systemic administration of pilocarpine (350 mg/kg, ip) and was allowed to proceed for 90 minutes. After the SE was terminated by sodium pentobarbital (30 mg/kg, ip), experimental rats were treated with selective COX‐2 inhibitor SC‐58125 (3 mg/kg, sc) twice daily for two consecutive days, and the ELISA was utilized to measure the total BDNF (both mature form and the precursor) in the hippocampus. This post‐SE time point was chosen for this study owing to the previous findings that COX‐2 inhibition for two days in rats was sufficient to completely block the PGE_2_ elevation and prevent the neuronal loss in the hippocampus after seizures.^31^ Here, we found that a 90‐minute episode of SE was able to induce the total BDNF protein levels for more than five times (Figure 2A). However, systemic treatment with SC‐58125 significantly decreased seizure‐induced BDNF production in the hippocampus when compared to the vehicle treatment (Figure 2A). It should also be noted that this administration regimen of the COX‐2 inhibitor did not completely abolish the SE‐induced BDNF elevation in the hippocampus, indicated by the significant difference between the two COX‐2 inhibitor treatment groups (*P* < .05, Figure 2A).

PGE_2_ is a dominant prostaglandin product of COX‐2 within the brain and is considered as a chief executor of COX‐2‐mediated inflammatory processes in a number of chronic brain conditions, such as strokes, seizures, neurodegenerative diseases, and brain tumors.^4^, ^32^, ^33^ To investigate whether PGE_2_ directly regulates BDNF synthesis and signaling, we treated the rat primary hippocampal cells (DIV14) with 16,16‐dimethyl PGE_2_ (dmPGE_2_)—a stable form of PGE_2_ for longer drug exposure—overnight and measured the BDNF levels in the culture medium using ELISA. We found that PGE_2_ treatment increased the levels of BDNF secreted by these primary hippocampal cells in a concentration‐dependent manner when compared to the control (Figure 2B). These in vitro and in vivo findings reveal that COX‐2 enzymatic activity is necessary for BDNF production in the hippocampus following SE, and PGE_2_ alone is sufficient to stimulate the synthesis of BDNF by the hippocampal cells.

### 3.3 EP2 receptor inhibition impairs BDNF expression in the hippocampus after SE

Among the four PGE_2_ receptor subtypes, EP2 is coupled to Gα_s_ and regulates a wide range of pathophysiological events in the brain via initiating cAMP signaling pathways.^4^, ^26^, ^33^, ^34^ It is has been well known that BDNF expression can be regulated by a number of transcription factors, among which the cAMP response element‐binding protein (CREB) is the most studied and can be activated by protein kinase A (PKA) upon cAMP binding^35^; the increased BDNF via TrkB in turn can stimulate CREB phosphorylation and activation via a calcium/calmodulin‐dependent kinase IV (CaMKIV).^36^ However, whether EP2 receptor contributes to cAMP pathway‐mediated BDNF upregulation and TrkB signaling remains unknown. Therefore, we next wanted to determine whether PGE_2_ signaling via EP2 receptor is involved in seizure‐induced BDNF/TrkB signaling following SE.

**FIGURE 3:**
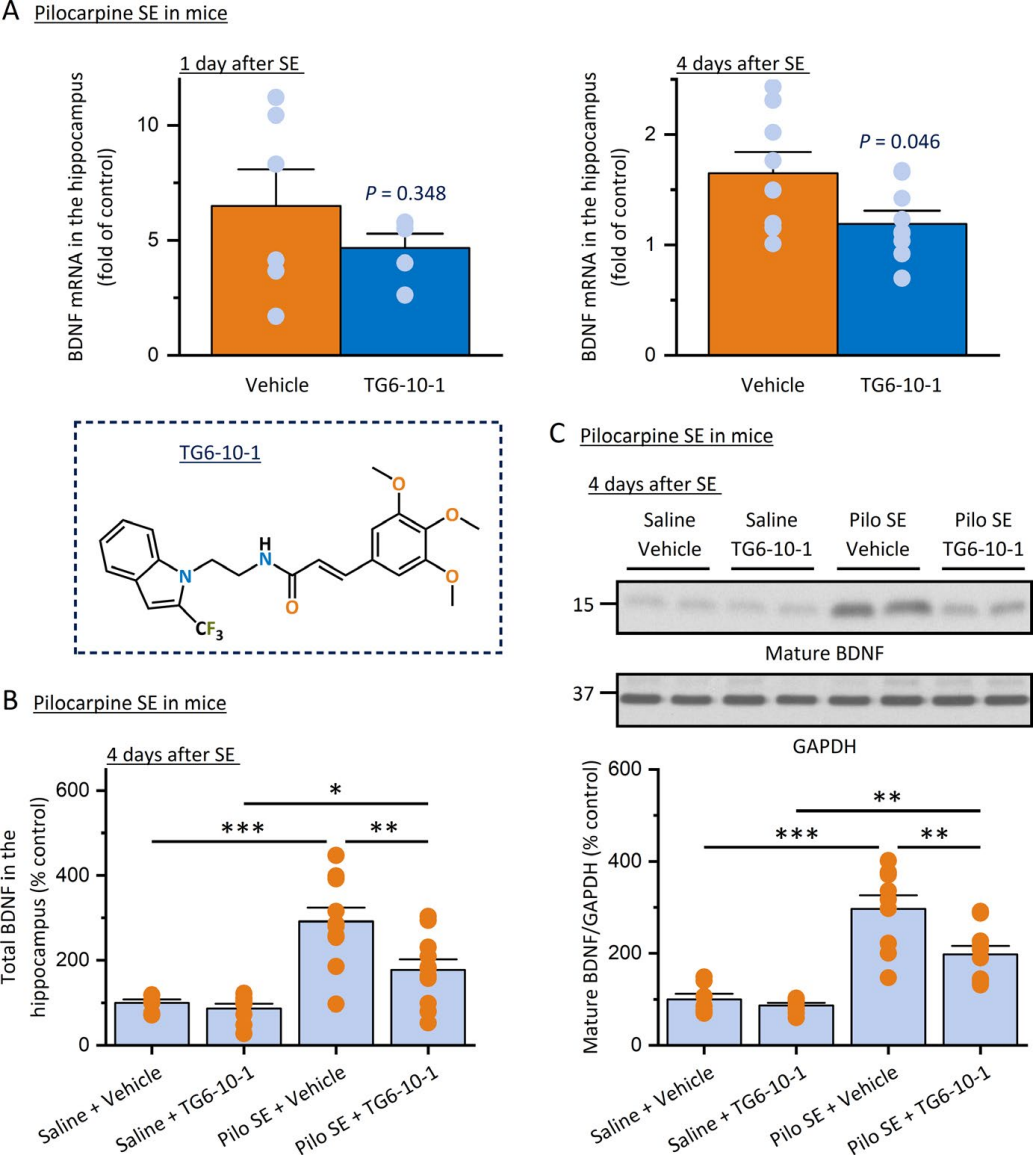
EP2 receptor inhibition blocks BDNF production after pilocarpine SE in mice. A, SE was induced in mice by pilocarpine (280 mg/kg, ip), allowed for 60 min, and terminated by sodium pentobarbital (30 mg/kg, ip). Mice were then treated by EP2 receptor antagonist TG6‐10‐1 (5 mg/kg, ip) twice daily. mRNA of BDNF in the hippocampus was measured by qPCR 1 d or 4 d after SE. The seizure‐induced BDNF mRNA was decreased by TG6‐10‐1 treatment at 1 day (N = 5‐6, *P* = .348 compared to vehicle treatment, unpaired t test) and 4 d (N = 8‐9, *P* = .046 compared to vehicle treatment, unpaired t test) after SE. The chemical structure of EP2 antagonist TG6‐10‐1 is displayed. B, The BDNF protein in the hippocampus was measured by ELISA 4 d after pilocarpine SE in mice. TG6‐10‐1 treatment prevented SE‐induced BDNF at the protein level (N = 8‐9, *P < .05; **P < .01; ***P < .001, one‐way ANOVA with post hoc Bonferroni's multiple comparisons test). C, The BDNF protein in the hippocampus was also measured by Western blot analysis. Two representative samples from each of the 4 experimental groups are shown on the blots. The immunoblots were scanned, and the band intensities were quantified using ImageJ. The relative protein levels of mature BDNF were normalized to GAPDH and then to their basal levels. TG6‐10‐1 treatment prevented SE‐induced mature BDNF (N = 8‐9, **P < .01; ***P < .001, one‐way ANOVA and post hoc Bonferroni's multiple comparisons test). All data shown as mean + SEM

SE was first induced in mice by systemic injection of pilocarpine (280 mg/kg, ip) and terminated by sodium pentobarbital (30 mg/kg, ip) 60 minutes later. Mice were then treated by our recently developed selective EP2 receptor antagonist TG6‐10‐1 (5 mg/kg, ip) twice daily. The mRNA levels of BDNF in the hippocampus were measured by qPCR 1 day and 4 days after SE, as we formerly reported that the post‐SE treatment with this EP2 antagonist significantly decreased the induction of a number of pro‐inflammatory cytokines and gliosis markers at 4 days—but not 1 day—after pilocarpine SE.^29^ In line with these previous findings, we found that the TG6‐10‐1‐treated mice only showed a trend of decrease in hippocampal BDNF mRNA expression at 1 day after SE when compared to their vehicle‐treated peers. However, at 4 days after SE, TG6‐10‐1 treatment nearly fully prevented the BDNF mRNA induction in the hippocampus (Figure 3A). Results from ELISA further revealed that the total BDNF protein level in the hippocampus was increased almost threefold at 4 days after pilocarpine SE when compared to the saline‐treated animals. However, systemic treatment with TG6‐10‐1 largely decreased SE‐induced BDNF in the hippocampus by nearly 60% compared to vehicle‐treated mice, measured also at 4 days after SE (Figure 3B). In line, the Western blot analysis revealed that the mature form of BDNF was increased about threefold in the hippocampus four days after pilocarpine SE; TG6‐10‐1 treatment considerably decreased SE‐induced mature BDNF in the hippocampus by about 50% when compared to vehicle treatment (Figure 3C). It should also be noted that this administration regimen of the EP2 antagonist did not completely eliminate the SE‐induced BDNF protein increase in the hippocampus as either total or mature form, shown by the considerable difference between the two TG6‐10‐1 treatment groups (*P* < .05, Figure 3B; *P* < .01, Figure 3C).

**FIGURE 4:**
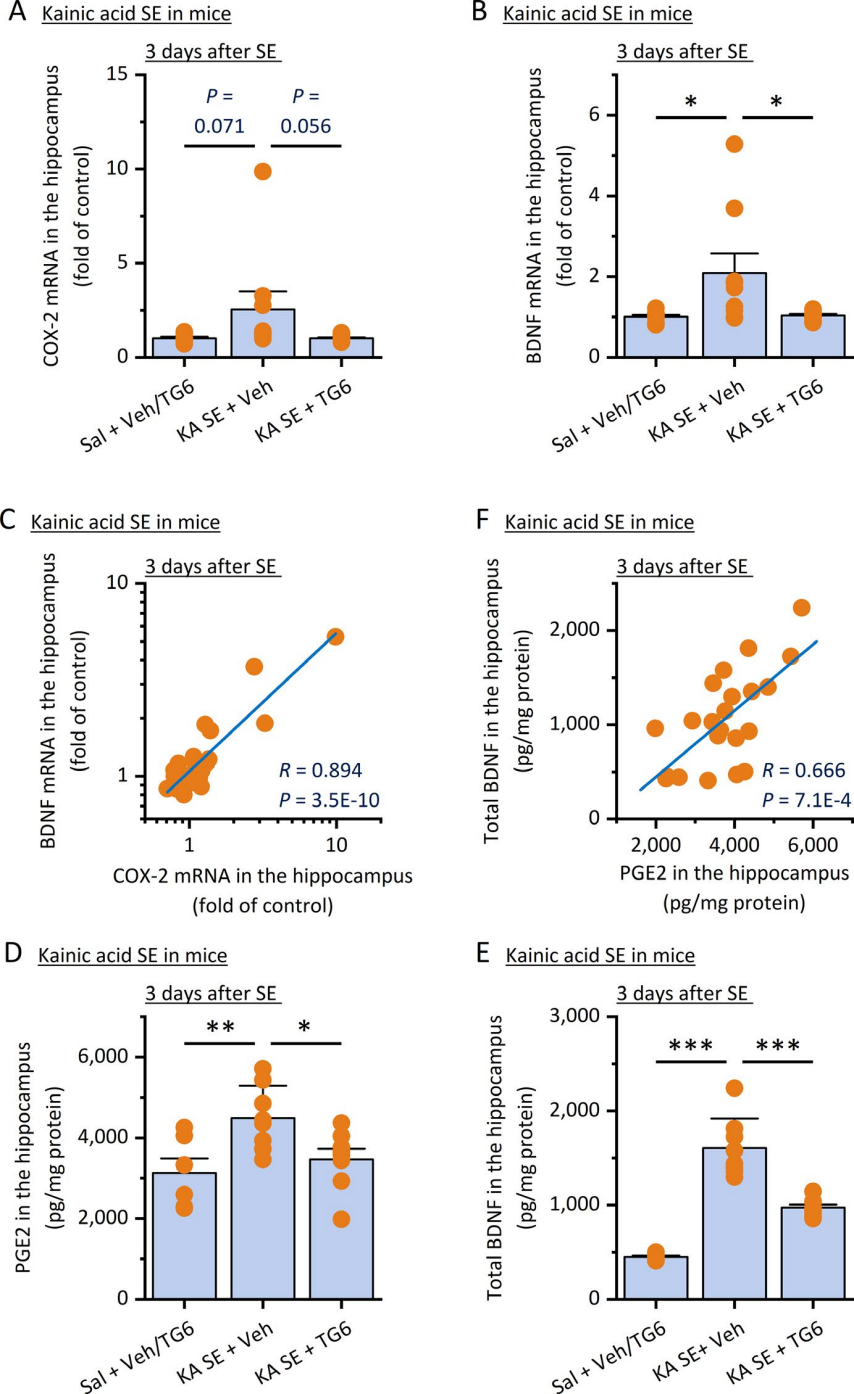
EP2 inhibition reduces BDNF production after kainic acid SE in mice. SE was induced in mice by kainic acid (KA, 30 mg/kg, ip), allowed for 60 min, and interrupted by diazepam (10 mg/kg, ip). Mice were then treated with TG6‐10‐1 (5 mg/kg, ip) twice daily for 3 d. The hippocampal expression of COX‐2 (A) and BDNF (B) was measured by qPCR for their mRNA levels and compared (N = 8‐10, *P < .05, one‐way ANOVA with post hoc Bonferroni's multiple comparisons test). Data are shown as mean + SEM. C, A positive correlation between COX‐2 and BDNF mRNA levels in the hippocampus was uncovered by Pearson correlation coefficient analysis (*R* = .894, *P* = 3.5E‐10). The levels of PGE_2_ (D) and total BDNF protein (E) in the hippocampus were also measured by ELISA. TG6‐10‐1 reduced both levels of PGE_2_ and total BDNF protein in the hippocampus (N = 6‐8, *P < .05; **P < .01; ***P < .001, one‐way ANOVA with post hoc Bonferroni's multiple comparisons test). Data are shown as mean + SEM. F, A positive correlation between PGE_2_ and total BDNF protein levels in the hippocampus was revealed by Pearson correlation coefficient analysis (*R* = .666, *P* = 7.1E‐4). Note that data from the two saline control groups did not show any significant difference to each other and thus were pooled for comparisons to the SE groups

Though pilocarpine is a commonly used chemoconvulsant for seizure induction in animals, it would be important to validate these findings derived from pilocarpine models using another proconvulsive agent to avoid any model‐specific results and conclusions. We next examined the effect of EP2 inhibition on BDNF expression in the hippocampus after SE triggered by kainic acid. Young adult mice were systemically treated with kainic acid (30 mg/kg, ip) to induce SE for 60 minutes, which was interrupted by diazepam (10 mg/kg, ip). Animals were then treated with EP2 antagonist TG6‐10‐1 (5 mg/kg, ip) twice daily, and the BDNF expression was assessed by qPCR and ELISA 3 days after SE. The 3‐day post‐SE time point was chosen for this study because we previously reported that the treatment with this EP2 antagonist showed significant anti‐inflammatory effects and other benefits, evaluated 3 days after kainic acid‐induced SE in mice.^24^ In line with the findings in pilocarpine models, a one‐hour episode of SE by kainic acid showed an evident trend of increase in hippocampal COX‐2 mRNA levels at 3 days after SE (*P* = .071, Figure 4A) and was able to double the BDNF mRNA expression in the hippocampus (*P* < .05, Figure 4B). However, TG6‐10‐1 treatment afforded a trend in decreasing the COX‐2 mRNA expression (*P* = .056, Figure 4A) and completely prevented the induction of BDNF mRNA in the hippocampus 3 days after SE when compared to the vehicle treatment (*P* < .05, Figure 4B). Interestingly, there was a positive correlation between COX‐2 and BDNF mRNA expression levels in the hippocampus (*R* = .894, Figure 4C). The ELISA results revealed that TG6‐10‐1 treatment for three consecutive days was also able to largely prevent the seizure‐induced production of PGE_2_ (Figure 4D) and the total BDNF (Figure 4E) in the hippocampus. Consistently, Pearson's analysis revealed a strong positive correlation between PGE_2_ and BDNF protein levels in the hippocampus (*R* = .666, Figure 4F). These findings from the three different animal models of SE together suggest that EP2 receptor might be a dominant PGE_2_ receptor that mediates COX‐2‐directed BDNF synthesis in the hippocampus following prolonged seizures.

### 3.4 PGE_2_ signaling via EP2 regulates CREB/BDNF/TrkB signaling following SE

**FIGURE 5:**
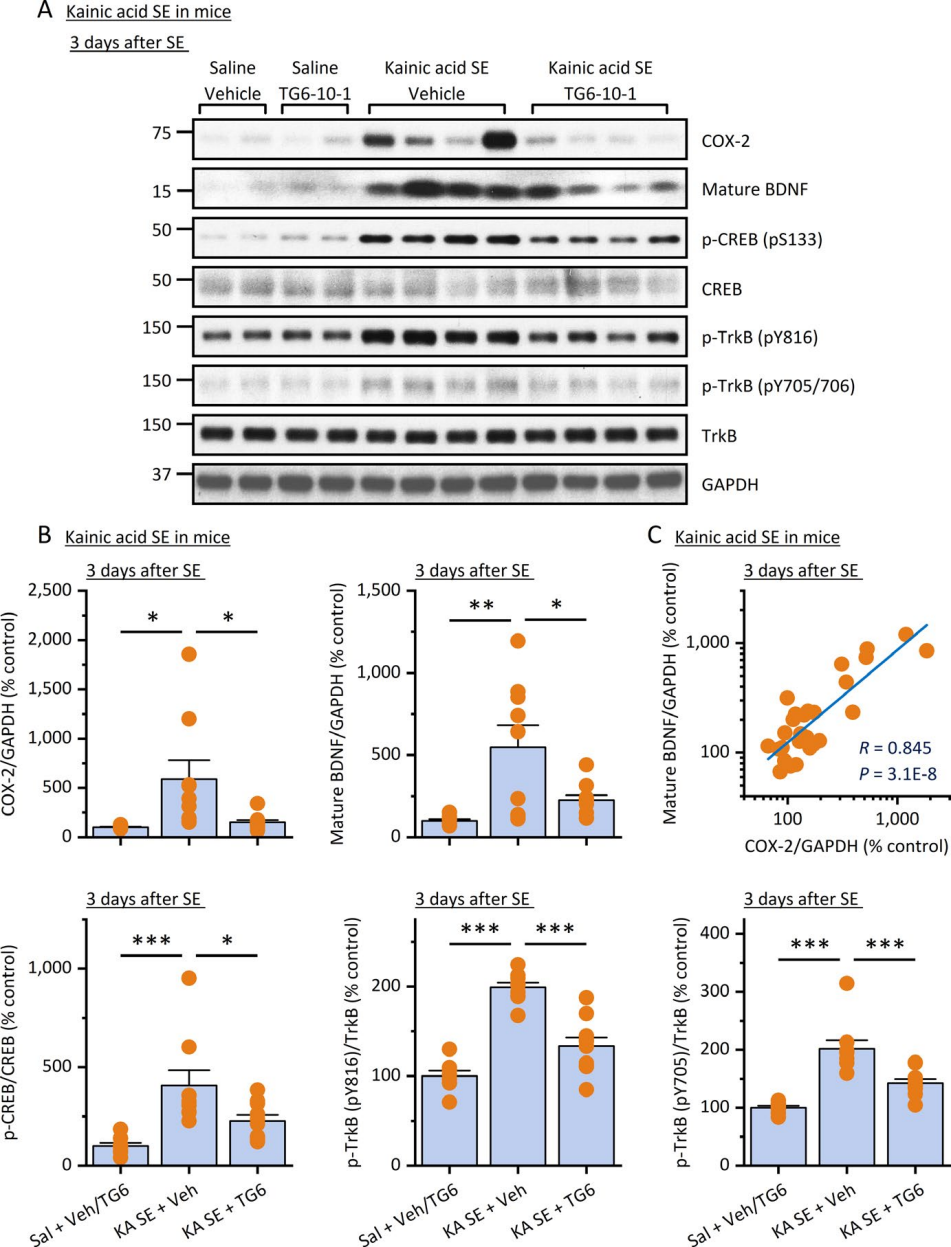
EP2 inhibition decreases BDNF/TrkB signaling following SE. A, Three days after kainic acid (KA) SE in mice, the COX‐2, mature BDNF, and activated CREB (pS133) and TrkB (pY816 and pY705/706) were measured by Western blot analysis. Representative samples from each of the 4 treatment groups are shown on the blots. B, The immunoblots were scanned, and the band intensities were quantified using ImageJ. The relative protein levels of COX‐2 and mature BDNF were normalized to GAPDH and then to their basal levels; the phosphorylated forms of CREB and TrkB were normalized to their total proteins and then to their basal levels. TG6‐10‐1 treatment prevented SE‐induced COX‐2, mature BDNF, and the phosphorylated forms of CREB and TrkB (N = 8‐10, *P < .05; **P < .01; ***P < .001 compared with control, one‐way ANOVA and post hoc Bonferroni's multiple comparisons test). All data are shown as mean + SEM. Note that data from the two saline control groups did not show any significant difference to each other and thus were pooled for comparisons to the SE groups. C, A positive correlation between COX‐2 and mature BDNF levels in the hippocampus was identified by Pearson correlation coefficient analysis (*R* = .845, *P* = 3.1E‐8)

We next used Western blot analysis to further examine the protein expression of COX‐2 and BDNF in the hippocampus after kainic acid‐induced SE in mice (Figure 5A). We found that both COX‐2 and mature BDNF were increased 5‐6 times in the hippocampus at the protein level three days after kainic acid SE (Figure 5B). In line, TG6‐10‐1 treatment nearly completely prevented the induction of COX‐2 and mature BDNF in the hippocampus following kainic acid SE in mice when compared to vehicle treatment (Figure 5B), and their expression levels showed a strong correlation revealed by Pearson's analysis (*R* = .845, Figure 5C). As a Gα_s_‐coupled receptor, the activation of EP2 can stimulate cAMP synthesis, leading to a variety of intracellular effects mediated by two cAMP‐binding effector proteins: cAMP‐dependent protein kinase (PKA) and exchange protein directly activated by cAMP (EPAC).^34^ Upon cAMP binding, the activated PKA is translocated to the nucleus, where it phosphorylates transcription factors responsive to cAMP such as the cAMP response element‐binding (CREB) protein. In line with the previous finding that seizure activity can activate CREB,^37^ our results show that kainic acid‐induced SE significantly increased the phosphorylation of CREB (pS133) in the hippocampus without altering the expression of total CREB (Figure 5A). However, this activated form of CREB was largely reduced by systemic treatment with TG6‐10‐1 (Figure 5B). EP2 inhibition by TG6‐10‐1 also decreased SE‐induced TrkB activation in the hippocampus, demonstrated by a significant reduction in phosphorylated forms of TrkB (both pY816 and pY705/706), detected 3 days after SE (Figure 5A); however, TG6‐10‐1 treatment did not alter the total TrkB expression (Figure 5B). These findings together suggest that EP2 receptor activation should largely be responsible for the COX‐2 activity‐mediated cAMP/CREB/BDNF/TrkB pathway in the hippocampus following SE. Therefore, the EP2 receptor inhibition might represent a novel strategy to suppress the BDNF/TrkB signaling‐mediated pathogenesis in the brain after prolonged seizures.

## 4 DISCUSSION

Utilizing pharmacological approaches and three classical chemoconvulsant animal models, the present study specifically aimed to investigate the role of COX/PGES/PGE_2_/EP signaling axis in the regulation of BDNF/TrkB signaling following prolonged seizures. We discovered that COX‐2, mPGES‐1 and BDNF were rapidly upregulated in the hippocampus at both mRNA and protein levels in a very similar time‐dependent manner following SE onset but with COX‐2 leading BDNF and mPGES‐1 temporally as well as quantitatively. Based on our findings that COX‐2 inhibition by compound SC‐58125 prevented BDNF elevation in the hippocampus following SE and that PGE_2_ alone was sufficient to directly stimulate hippocampal cells to secrete BDNF in primary cultures, we hypothesized that a cAMP signaling pathway initiated by PGE_2_ might be involved in BDNF/TrkB signaling in the hippocampus following SE. Using a novel bioavailable and brain‐permeable small‐molecule antagonist that we recently developed to suppress inflammation within the brain, TG6‐10‐1, we found that pharmacological inhibition of the Gα_s_‐coupled PGE_2_ receptor EP2 decreased SE‐triggered BDNF/TrkB activation in the hippocampus. Our findings established the COX‐2/PGE_2_/EP2 axis as an important upstream pathway that is immediately triggered by prolonged SE and plays an essential role in the regulation of hippocampal BDNF/TrkB signaling prior to epileptogenesis.

### 4.1 COX‐2 as a conventional target

Mounting evidence from numerous preclinical and clinical studies indicates that inflammation in the CNS is an intrinsic characteristic of the neuronal hyperexcitability in epilepsy of various etiologies.^38^, ^39^ The pro‐inflammatory mediators that have been reported to contribute to the precipitation of acute seizures and potentially acquired epileptogenesis include interleukin‐1β (IL‐1β), IL‐6, tumor necrosis factor‐α (TNF‐α), transforming growth factor‐β (TGF‐β), high mobility group box 1 (HMGB1), and Toll‐like receptor 4 (TLR4).^3^, ^40^ The inducible COX isozyme, COX‐2, sits atop another large inflammatory signaling axis associated with epileptic seizures. Treatment with selective COX‐2 inhibitor celecoxib decreased the BDNF induction in the hippocampal dentate gyrus in rats that were treated by kainic acid.^21^ As a major prostanoid product of COX‐2 in the brain, PGE_2_ often functions as a chief executor of COX‐2 cascade‐dictated neuropathogenesis.^4^, ^32^ SE induced by electrical stimulation substantially increased the expression of COX‐2, leading to the synthesis of PGE_2_ in the hippocampal subregions in rats; the COX inhibition by flurbiprofen decreased the levels of PGE_2_ as well as SE‐induced BDNF in the hippocampus.^41^ In line, we found that the selective COX‐2 inhibition by SC‐58125 largely prevented the production of hippocampal BDNF in rats experienced pilocarpine‐induced SE. It seems that inhibiting COX‐2 might represent a strategy to prevent the elevated BDNF/TrkB signaling after SE.

### 4.2 EP2 as an emerging target

In addition to PGE_2_, COX‐2 enzymatic activity also leads to the syntheses of the other four prostanoids—that is, PGD_2_, PGF_2α_, PGI_2_, and TXA_2_—that can mediate a number of pro‐ and anti‐inflammatory effects depending on their downstream receptors involved.^4^ Therefore, targeting COX‐2 for controlling BDNF/TrkB signaling after SE may not provide sufficient therapeutic specificity and could theoretically cause untoward effects. The past two decades also witnessed the growing recognition of the undesirable effects of selective COX‐2 inhibitors on microvessels, leading to enormous risks for severe cerebrovascular and cardiovascular incidents.^42^ The finding that post‐SE treatment with our newly developed bioavailable brain‐permeable small‐molecule antagonist TG6‐10‐1 substantially impaired the seizure‐triggered BDNF/TrkB signaling suggests that the upregulation of BDNF production by COX‐2 should largely be attributed to the EP2 receptor activation by PGE_2_. Therefore, targeting the EP2 receptor for abnormal BDNF/TrkB signaling provides an alternative strategy to COX‐2 inhibition with higher specificity. However, it should also be noted that neither COX‐2 inhibition by SC‐58125 nor EP2 antagonism by TG6‐10‐1 was able to completely block the protein elevation of hippocampal BDNF after SE (Figures 2A,3B,C and 4E) and the consequent activation of CREB and TrkB (Figure 5), thought its mRNA increase can be fully prevented by inhibiting EP2 with TG6‐10‐1 (Figures 3A and 4B). These seemingly contradicting outcomes from the transcriptional and translational measures might suggest that some mechanisms that are independent of the COX‐2/PGE_2_/EP2 axis could also be involved in the seizure‐induced BDNF/TrkB signaling.

### 4.3 Using multiple models of SE

Both pilocarpine and kainic acid are commonly used to induce prolonged seizures in experimental mice and rats. These two animal models of SE share some important commonalities that simulate human SE conditions: (a) without intervention SE induced by pilocarpine and kainic acid can sustain for hours; (b) SE is often followed by a latent period without animals showing obvious seizure‐like activities for days to weeks; (c) prolonged SE (>45 minutes) causes long‐lasting inflammation and profound neuronal injury within the brain; and (d) survival animals after SE gradually develop unprovoked seizures that often recur with increasing frequency and without remission.^43^, ^44^, ^45^ However, the proconvulsant effect of pilocarpine is derived from its capability to activate the muscarinic acetylcholine receptor subtype M1^46^; whereas kainic acid induces experimental seizures via acting on the glutamate receptor subtype GluK1 in interneurons and GluK2 in principal neurons.^47^, ^48^ Thus, these two common chemoconvulsants are very likely to have different central and peripheral effects. As such, more than 95% of gene expression alterations in dentate granule cells of the hippocampus detected after kainic acid, pilocarpine, or electrically triggered SE are quite different,^49^ highlighting the fundamental discrepancies among these preclinical models and attesting to the value of using multiple animal models in proof‐of‐concept studies. In the present study, both kainic acid and pilocarpine‐induced prolonged seizures caused a significant upregulation of BDNF/TrkB signaling in the hippocampus, suggesting that the expression change of BDNF was immediately caused by seizure activities but not directly by the chemoconvulsants themselves. EP2 inhibition by TG6‐10‐1 was able to largely prevent the BDNF induction in the hippocampus following both kainic acid and pilocarpine‐induced prolonged seizures, indicating that these findings are unlikely model‐specific; using both mice and rats in this study lowered the likelihood that our discoveries are species‐specific. Importantly, these results together also exclude a direct effect of TG6‐10‐1 on either glutamate system or acetylcholine system; rather, the anti‐BDNF/TrkB effect of EP2 inhibition was likely derived from a repressive action on cAMP production and its downstream effectors. As a limitation, due to the scope of this study, we did not verify the involvement of EP2 receptor in hippocampal BDNF induction and the consequent phosphorylation of CREB and TrkB following SE triggered by electrical stimulation, in which COX activity was also required for the BDNF synthesis.^41^

### 4.4 Selective EP2 inhibition

EP2 and EP4 receptors share common natural ligand (ie, PGE_2_) and are both coupled to Gα_s_ mediating cAMP signaling pathways. We previously reported that the compound TG6‐10‐1, as a competitive antagonist, was more than 600‐fold selective for EP2 over EP4.^23^ With systemic administration (5 mg/kg, ip) in mice, TG6‐10‐1 should not show any inhibition on EP4 receptor in vivo, as its maximal concentration in the brain was well below its EP4 Schild *K*_B_ value.^30^ Therefore, its inhibitory effect on BDNF production and TrkB activation in the brain should mainly be attributed to its action on the EP2 receptor subtype. However, selectively blocking EP2 receptor by TG6‐10‐1 after SE did not fully prevent the elevation of BDNF protein expression in the hippocampus (Figures 3B,C and 4E), nor did it completely revert the consequent phosphorylation of CREB and TrkB receptor in the same brain area after SE (Figure 5). Thus, it is likely that EP4, as the other Gα_s_‐coupled PGE_2_ receptor subtype, might also contribute to the seizure‐promoted BDNF elevation. Future studies should be directed to investigate this possibility in order to fully understand the molecular mechanisms whereby COX‐2/PGE_2_ axis regulates TrkB signaling.

Furthermore, we previously revealed that even at a very high concentration (10 µmol/L), TG6‐10‐1 had negligible effect on the enzymatic activities of COX‐1 (~7% inhibition) and COX‐2 (~14% inhibition).^23^ Thus, it is very unlikely that TG6‐10‐1 decreased hippocampal BDNF expression after SE via directly inhibiting the two COX enzymes. However, EP2 receptor inhibition by TG6‐10‐1 led to a significant reduction in the expression of a number of important pro‐inflammatory mediators including COX‐2 and PGE_2_ within the brain after prolonged seizures (Figures 4A,D and 5A,B).^23^, ^24^, ^50^ Therefore, the reductive effects of TG6‐10‐1 on COX‐2 expression and PGE_2_ production might indirectly contribute to its inhibitory effect on hippocampal BDNF expression following prolonged seizures.

## 5 CONCLUSIONS

Post‐SE treatment with TG6‐10‐1 decreased the phosphorylation of CREB, which was used to indicate its activation in the hippocampus after SE. It is well known that the activation of CREB can upregulate BDNF expression and the consequent TrkB activation,^35^ which in turn can increase CREB phosphorylation and activation through a CaMK‐dependent mechanism.^36^ However, whether Gα_s_‐coupled EP2 receptor regulates BDNF/TrkB signaling following SE via activating the cAMP/PKA/CREB pathway and whether inhibiting CREB activity can block seizure‐promoted BDNF/TrkB signaling remain to be determined for the future studies. Nonetheless, our findings suggest that COX‐2 via PGE_2_/EP2 signaling regulates the hippocampal BDNF/TrkB pathway following prolonged seizures. Thus, EP2 inhibition by our brain‐permeable antagonists such as TG6‐10‐1 might provide a novel strategy to suppress the abnormal TrkB activity during acquired epileptogenesis.

## CONFLICT OF INTEREST

None of the authors has any conflict of interest to disclose. We confirm that we have read the Journal's position on issues involved in ethical publication and affirm that this report is consistent with those guidelines.

## References

[1] Chen Z , Brodie MJ , Liew D , Kwan P . Treatment outcomes in patients with newly diagnosed epilepsy treated with established and new antiepileptic drugs: a 30‐year Longitudinal Cohort Study. JAMA Neurol. 2018;75:279–86. 29279892 10.1001/jamaneurol.2017.3949 PMC5885858

[2] Perucca P , Gilliam FG . Adverse effects of antiepileptic drugs. Lancet Neurol. 2012;11:792–802. 22832500 10.1016/S1474-4422(12)70153-9

[3] Dey A , Kang X , Qiu J , Du Y , Jiang J . Anti‐inflammatory small molecules to treat seizures and epilepsy: from bench to bedside. Trends Pharmacol Sci. 2016;37:463–84. 27062228 10.1016/j.tips.2016.03.001 PMC5064857

[4] Yu Y , Nguyen DT , Jiang J . G protein‐coupled receptors in acquired epilepsy: druggability and translatability. Prog Neurogibol. 2019;183:101682. 10.1016/j.pneurobio.2019.101682 PMC6927250 31454545

[5] Binder DK , Croll SD , Gall CM , Scharfman HE . BDNF and epilepsy: too much of a good thing? Trends Neurosci. 2001;24:47–53. 11163887 10.1016/s0166-2236(00)01682-9

[6] Tongiorgi E , Armellin M , Giulianini PG , Bregola G , Zucchini S , Paradiso B , et al. Brain‐derived neurotrophic factor mRNA and protein are targeted to discrete dendritic laminas by events that trigger epileptogenesis. J Neurosci. 2004;24:6842–52. 15282290 10.1523/JNEUROSCI.5471-03.2004 PMC6729709

[7] Minichiello L . TrkB signalling pathways in LTP and learning. Nat Rev Neurosci. 2009;10:850–60. 19927149 10.1038/nrn2738

[8] Takahashi M , Hayashi S , Kakita A , Wakabayashi K , Fukuda M , Kameyama S , et al. Patients with temporal lobe epilepsy show an increase in brain‐derived neurotrophic factor protein and its correlation with neuropeptide Y. Brain Res. 1999;818:579–82. 10082852 10.1016/s0006-8993(98)01355-9

[9] Jankowsky JL , Patterson PH . The role of cytokines and growth factors in seizures and their sequelae. Prog Neurogibol. 2001;63:125–49. 10.1016/s0301-0082(00)00022-8 11124444

[10] Wyneken U , Smalla K‐H , Marengo JJ , Soto D , de la Cerda A , Tischmeyer W , et al. Kainate‐induced seizures alter protein composition and N‐methyl‐D‐aspartate receptor function of rat forebrain postsynaptic densities. Neuroscience. 2001;102:65–74. 11226670 10.1016/s0306-4522(00)00469-3

[11] Xu B , Michalski B , Racine RJ , Fahnestock M . The effects of brain‐derived neurotrophic factor (BDNF) administration on kindling induction, Trk expression and seizure‐related morphological changes. Neuroscience. 2004;126:521–31. 15183502 10.1016/j.neuroscience.2004.03.044

[12] Thomas AX , Cruz Del Angel Y , Gonzalez MI , Carrel AJ , Carlsen J , Lam PM , et al. Rapid increases in proBDNF after pilocarpine‐induced status epilepticus in mice are associated with reduced proBDNF cleavage machinery. eNeuro. 2016;3(1):ENEURO.0020‐15.2016. 10.1523/ENEURO.0020-15.2016 PMC4814566 27057559

[13] Binder DK , Routbort MJ , Ryan TE , Yancopoulos GD , McNamara JO . Selective inhibition of kindling development by intraventricular administration of TrkB receptor body. J Neurosci. 1999;19:1424–36. 9952419 10.1523/JNEUROSCI.19-04-01424.1999 PMC6786043

[14] He XP , Minichiello L , Klein R , McNamara JO . Immunohistochemical evidence of seizure‐induced activation of trkB receptors in the mossy fiber pathway of adult mouse hippocampus. J Neurosci. 2002;22:7502–8. 12196573 10.1523/JNEUROSCI.22-17-07502.2002 PMC6757988

[15] He XP , Kotloski R , Nef S , Luikart BW , Parada LF , McNamara JO . Conditional deletion of TrkB but not BDNF prevents epileptogenesis in the kindling model. Neuron. 2004;43:31–42. 15233915 10.1016/j.neuron.2004.06.019

[16] He XP , Pan E , Sciarretta C , Minichiello L , McNamara JO . Disruption of TrkB‐mediated phospholipase Cgamma signaling inhibits limbic epileptogenesis. J Neurosci. 2010;30:6188–96. 20445044 10.1523/JNEUROSCI.5821-09.2010 PMC2872928

[17] Liu G , Gu B , He X‐P , Joshi R , Wackerle H , Rodriguiz R . Transient inhibition of TrkB kinase after status epilepticus prevents development of temporal lobe epilepsy. Neuron. 2013;79:31–8. 23790754 10.1016/j.neuron.2013.04.027 PMC3744583

[18] Gu B , Huang YZ , He XP , Joshi RB , Jang W , McNamara JO . A peptide uncoupling BDNF receptor TrkB from phospholipase Cgamma1 prevents epilepsy induced by status epilepticus. Neuron. 2015;88:484–91. 26481038 10.1016/j.neuron.2015.09.032 PMC4636438

[19] McNamara JO , Scharfman HE . Temporal lobe epilepsy and the BDNF receptor, TrkB In: Noebels JL, Avoli M, Rogawski MA, Olsen RW, Delgado‐Escueta AV, editors. Jasper's basic mechanisms of the epilepsies, 4th ed Bethesda, MD: National Center for Biotechnology Information (US); 2012. 22787630

[20] Lin TW , Harward SC , Huang YZ , McNamara JO . Targeting BDNF/TrkB pathways for preventing or suppressing epilepsy. Neuropharmacology. 2020;167:107734. 31377199 10.1016/j.neuropharm.2019.107734 PMC7714524

[21] Gobbo OL , O'Mara SM . Post‐treatment, but not pre‐treatment, with the selective cyclooxygenase‐2 inhibitor celecoxib markedly enhances functional recovery from kainic acid‐induced neurodegeneration. Neuroscience. 2004;125:317–27. 15062975 10.1016/j.neuroscience.2004.01.045

[22] Jiang J , Ganesh T , Du Y , Quan Y , Serrano G , Qui M . Small molecule antagonist reveals seizure‐induced mediation of neuronal injury by prostaglandin E2 receptor subtype EP2. Proc Natl Acad Sci U S A. 2012;109:3149–54. 22323596 10.1073/pnas.1120195109 PMC3286971

[23] Jiang J , Quan Y , Ganesh T , Pouliot WA , Dudek FE , Dingledine R . Inhibition of the prostaglandin receptor EP2 following status epilepticus reduces delayed mortality and brain inflammation. Proc Natl Acad Sci U S A. 2013;110:3591–6. 23401547 10.1073/pnas.1218498110 PMC3587237

[24] Jiang J , Yu Y , Kinjo ER , Du Y , Nguyen HP , Dingledine R . Suppressing pro‐inflammatory prostaglandin signaling attenuates excitotoxicity‐associated neuronal inflammation and injury. Neuropharmacology. 2019;149:149–60. 30763657 10.1016/j.neuropharm.2019.02.011 PMC6486887

[25] Yu Y , Li L , Nguyen DT , Mustafa SM , Moore BM , Jiang J . Inverse agonism of cannabinoid receptor Type 2 confers anti‐inflammatory and neuroprotective effects following status epileptics. Mol Neurobiol. 2020;57:2830–45. 32378121 10.1007/s12035-020-01923-4 PMC7282534

[26] Quan Y , Jiang J , Dingledine R . EP2 receptor signaling pathways regulate classical activation of microglia. J Biol Chem. 2013;288:9293–302. 23404506 10.1074/jbc.M113.455816 PMC3611000

[27] Kang X , Qiu J , Li Q , Bell KA , Du Y , Jung DW , et al. Cyclooxygenase‐2 contributes to oxidopamine‐mediated neuronal inflammation and injury via the prostaglandin E2 receptor EP2 subtype. Sci Rep. 2017;7:9459. 28842681 10.1038/s41598-017-09528-z PMC5573328

[28] Qiu J , Li Q , Bell KA , Yao X , Du Y , Zhang E , et al. Small‐molecule inhibition of prostaglandin E receptor 2 impairs cyclooxygenase‐associated malignant glioma growth. Br J Pharmacol. 2019;176:1680–99. 30761522 10.1111/bph.14622 PMC6514294

[29] Jiang J , Yang MS , Quan Y , Gueorguieva P , Ganesh T , Dingledine R . Therapeutic window for cyclooxygenase‐2 related anti‐inflammatory therapy after status epilepticus. Neurobiol Dis. 2015;76:126–36. 25600211 10.1016/j.nbd.2014.12.032 PMC4408774

[30] Du Y , Kemper T , Qiu J , Jiang J . Defining the therapeutic time window for suppressing the inflammatory prostaglandin E2 signaling after status epilepticus. Expert Rev Neurother. 2016;16:123–30. 26689339 10.1586/14737175.2016.1134322 PMC5070609

[31] Takemiya T , Maehara M , Matsumura K , Yasuda S , Sugiura H , Yamagata K . Prostaglandin E2 produced by late induced COX‐2 stimulates hippocampal neuron loss after seizure in the CA3 region. Neurosci Res. 2006;56:103–10. 16837093 10.1016/j.neures.2006.06.003

[32] Andreasson K . Emerging roles of PGE2 receptors in models of neurological disease. Prostaglandins Other Lipid Mediat. 2010;91:104–12. 19808012 10.1016/j.prostaglandins.2009.04.003 PMC2846228

[33] Jiang J , Qiu J , Li Q , Shi Z . Prostaglandin E2 signaling: alternative target for glioblastoma? Trends Cancer. 2017;3:75–8. 28718447 10.1016/j.trecan.2016.12.002 PMC5518646

[34] Jiang J , Dingledine R . Prostaglandin receptor EP2 in the crosshairs of anti‐inflammation, anti‐cancer, and neuroprotection. Trends Pharmacol Sci. 2013;34:413–23. 23796953 10.1016/j.tips.2013.05.003 PMC4031445

[35] Xue W , Wang W , Gong T , Zhang H , Tao W , Xue L , et al. PKA‐CREB‐BDNF signaling regulated long lasting antidepressant activities of Yueju but not ketamine. Sci Rep. 2016;6:26331. 27197752 10.1038/srep26331 PMC4873804

[36] Finkbeiner S , Tavazoie SF , Maloratsky A , Jacobs KM , Harris KM , Greenberg ME . CREB: a major mediator of neuronal neurotrophin responses. Neuron. 1997;19:1031–47. 9390517 10.1016/s0896-6273(00)80395-5

[37] Lund IV , Hu Y , Raol YH , Benham RS , Faris R , Russek SJ , et al. BDNF selectively regulates GABAA receptor transcription by activation of the JAK/STAT pathway. Sci Signal. 2008;1:ra9. 18922788 10.1126/scisignal.1162396 PMC2651003

[38] Wilcox KS , Vezzani A . Does brain inflammation mediate pathological outcomes in epilepsy? Adv Exp Med Biol. 2014;813:169–83. 25012376 10.1007/978-94-017-8914-1_14 PMC4867105

[39] Koepp MJ , Arstad E , Bankstahl JP , Dedeurwaerdere S , Friedman A , Potschka H , et al. Neuroinflammation imaging markers for epileptogenesis. Epilepsia. 2017;58(Suppl 3):11–9. 28675560 10.1111/epi.13778

[40] Klein P , Dingledine R , Aronica E , Bernard C , Blümcke I , Boison D , et al. Commonalities in epileptogenic processes from different acute brain insults: do they translate? Epilepsia. 2018;59:37–66. 29247482 10.1111/epi.13965 PMC5993212

[41] Ajmone‐Cat MA , Iosif RE , Ekdahl CT , Kokaia Z , Minghetti L , Lindvall O . Prostaglandin E2 and BDNF levels in rat hippocampus are negatively correlated with status epilepticus severity: no impact on survival of seizure‐generated neurons. Neurobiol Dis. 2006;23:23–35. 16531049 10.1016/j.nbd.2006.01.010

[42] Grosser T , Yu Y , Fitzgerald GA . Emotion recollected in tranquility: lessons learned from the COX‐2 saga. Annu Rev Med. 2010;61:17–33. 20059330 10.1146/annurev-med-011209-153129

[43] Reddy DS , Kuruba R . Experimental models of status epilepticus and neuronal injury for evaluation of therapeutic interventions. Int J Mol Sci. 2013;14:18284–318. 24013377 10.3390/ijms140918284 PMC3794781

[44] Covolan L , Mello LE . Temporal profile of neuronal injury following pilocarpine or kainic acid‐induced status epilepticus. Epilepsy Res. 2000;39:133–52. 10759302 10.1016/s0920-1211(99)00119-9

[45] Loscher W . Animal models of seizures and epilepsy: past, present, and future role for the discovery of antiseizure drugs. Neurochem Res. 2017;42:1873–88. 28290134 10.1007/s11064-017-2222-z

[46] Bymaster FP , Carter PA , Yamada M , Gomeza J , Wess J , Hamilton SE , et al. Role of specific muscarinic receptor subtypes in cholinergic parasympathomimetic responses, in vivo phosphoinositide hydrolysis, and pilocarpine‐induced seizure activity. Eur J Neurosci. 2003;17:1403–10. 12713643 10.1046/j.1460-9568.2003.02588.x

[47] Mulle C , Sailer A , Pérez‐Otaño I , Dickinson‐Anson H , Castillo PE , Bureau I , et al. Altered synaptic physiology and reduced susceptibility to kainate‐induced seizures in GluR6‐deficient mice. Nature. 1998;392:601–5. 9580260 10.1038/33408

[48] Ben‐Ari Y . Kainate and Temporal Lobe Epilepsies: 3 decades of progress In: Noebels JL, Avoli M, Rogawski MA, Olsen RW, Delgado‐Escueta AV, editors. Jasper's basic mechanisms of the epilepsies. Bethesda, MD: National Center for Biotechnology Information (US); 2012. 22787646

[49] Dingledine R , Coulter DA , Fritsch B , Gorter JA , Lelutiu N , McNamara J , et al. Transcriptional profile of hippocampal dentate granule cells in four rat epilepsy models. Sci Data. 2017;4:170061. 28485718 10.1038/sdata.2017.61 PMC5423390

[50] Nagib MM , Yu Y , Jiang J . Targeting prostaglandin receptor EP2 for adjunctive treatment of status epilepticus. Pharmacol Ther. 2020;209:107504. 32088247 10.1016/j.pharmthera.2020.107504 PMC7222917

